# Quantifying the physical activity energy expenditure of commuters using a combination of global positioning system and combined heart rate and movement sensors

**DOI:** 10.1016/j.ypmed.2015.09.022

**Published:** 2015-12

**Authors:** Silvia Costa, David Ogilvie, Alice Dalton, Kate Westgate, Søren Brage, Jenna Panter

**Affiliations:** a MRC Epidemiology Unit and UKCRC Centre for Diet and Activity Research (CEDAR), School of Clinical Medicine, University of Cambridge, Cambridge, UK; b Norwich Medical School and UKCRC Centre for Diet and Activity Research (CEDAR), University of East Anglia, Norwich, UK; c MRC Epidemiology Unit, School of Clinical Medicine, University of Cambridge, Cambridge, UK

**Keywords:** Transportation, Exercise, GPS, Geographical Information Systems, Active commuting, Walking, Cycling

## Abstract

**Background:**

Active commuting may help to increase adults' physical activity levels. However, estimates of its energy cost are derived from a small number of studies which are laboratory-based or use self-reported measures.

**Methods:**

Adults working in Cambridge (UK) recruited through a predominantly workplace-based strategy wore combined heart rate and movement sensors and global positioning system (GPS) devices for one week, and completed synchronous day-by-day travel diaries in 2010 and 2011. Commuting journeys were delineated using GPS data, and metabolic intensity (standard metabolic equivalents; MET) was derived and compared between journey types using mixed-effects linear regression.

**Results:**

182 commuting journeys were included in the analysis. Median intensity was 1.28 MET for car journeys; 1.67 MET for bus journeys; 4.61 MET for walking journeys; 6.44 MET for cycling journeys; 1.78 MET for journeys made by car in combination with walking; and 2.21 MET for journeys made by car in combination with cycling. The value for journeys made solely by car was significantly lower than those for all other journey types (*p* < 0.04). On average, 20% of the duration of journeys incorporating any active travel (equating to 8 min) was spent in moderate-to-vigorous physical activity (MVPA).

**Conclusions:**

We have demonstrated how GPS and activity data from a free-living sample can be used simultaneously to provide objective estimates of commuting energy expenditure. On average, incorporating walking or cycling into longer journeys provided over half the weekly recommended activity levels from the commute alone. This may be an efficient way of achieving physical activity guidelines and improving population health.

## Introduction

Physical inactivity is the fourth leading cause of global mortality, and the World Health Organization, the United Nations and numerous national governments now view the promotion of physical activity as a public health priority (Department of Health, 2011, United Nations, 2011, World Health Organisation, 2000). Incorporating walking and cycling into commuting journeys is one way of increasing physical activity that may be more easily adopted and maintained in everyday life than some other forms of activity (Department of Health, 2011). Epidemiological studies suggest beneficial effects of active commuting on cardiovascular risk independent of other physical activities (Hamer & Chida, 2008), and modelling studies suggest that the health benefits of a shift towards active travel greatly outweigh the harms (Woodcock et al., 2009, Woodcock et al., 2013). However, accurate estimates of the metabolic cost associated with different commuting patterns in free-living conditions are required to quantify the health impacts of interventions aimed at changing travel behaviour (Shephard, 2008).

To derive such estimates, information on the intensity, duration and frequency of activities is required, but capturing this information is notoriously difficult. Self-reported measures are subject to recall and social desirability bias, and objective measures are often only available in small samples (Shephard, 2003). The physical activity compendium provides estimates of the metabolic cost (in metabolic equivalents, MET) for a range of different activities, sometimes from several studies. Estimates are given with and without assumptions about speed, gradient, and other factors which would influence metabolic cost, such as walking with or without a load. The compendium has been updated as new studies are published and new codes added (Ainsworth et al., 2000, Ainsworth et al., 2011). However, the source publications for the estimate of energy expenditure for walking to work are either old or based on self-reported measures which have been validated in laboratory studies (Taylor et al., 1978), whilst the estimate for cycling to work uses objective data from a single study with a relatively small sample (de Geus et al., 2007). Evidence from more studies would therefore give greater confidence in the compendium estimates for these activities. These limitations equally apply to the quantification of energy expenditure of travel behaviours at the lower end of the intensity spectrum, such as travelling by public transport or car (Bandyopadhyay & Chattopadhyay, 1980). Thus, whilst walking or cycling parts of a longer journey made by motor vehicle may be encouraged for health reasons, the extent to which these active stages of the journey contribute to overall energy expenditure in an otherwise sedentary journey is uncertain.

The widespread availability of global positioning system (GPS) devices and physical activity monitors means that objective data can now be collected in free-living conditions without the need for direct observation (Krenn et al., 2011). We therefore aimed use this combination of measures to quantify the metabolic cost of physical activity associated with the use of different modes and combinations of modes of transport for commuting.

## Methods

### Study setting and participant recruitment

The Commuting and Health in Cambridge study protocol and recruitment procedures have been reported elsewhere (Ogilvie et al., 2010). Briefly, 1164 adults aged 16 years and over who lived within 30 km of the centre of Cambridge, UK and travelled to work in the city were recruited in 2009, predominantly through workplaces via emails, recruitment stands and advertisements. The city of Cambridge lies approximately 80 km northeast of London and has a generally flat topography, a large student population and the traffic congestion in its historic city centre. The surrounding rural area includes smaller towns as well as a large number of small settlements.

### Data collection

Further information on the sample and data collection methods are given elsewhere (Panter et al., 2014). Briefly, in 2009 and 2010, participants completed a questionnaire to assess personal characteristics and travel behaviours, and a subsample were invited to wear accelerometers (n = 714) (ActiGraph, Pensacola, FL, USA) for seven days (Yang et al., 2012). One year later, the subsample who had provided valid accelerometer data (n = 550) were invited to complete a follow-up questionnaire and travel diary and to wear combined heart rate and movement sensors (Actiheart; CamNtech Ltd, Cambridge, UK) and GPS (BT-Q1000X; QStarz, Taipei, Taiwan) devices. Each participant attended an appointment with a research assistant where they gave written informed consent, had their height and weight measured for the computation of body mass index (BMI) (weight divided by height squared), and were asked to wear both devices for seven consecutive days and to complete the travel diary over the same period. Ethical approval was obtained from the Hertfordshire Research Ethics Committee (reference numbers 09/H0311/116 and 10/H0311/65).

#### Objective measures

The Actiheart combined acceleration and heart rate sensor (AccHR) is a lightweight (10g) waterproof device that clips onto two standard electrodes attached to the chest. It has been shown to be a valid and reliable tool to assess activity, providing a more accurate assessment of energy expenditure than accelerometry alone (Brage et al., 2005). The devices were set to continuously collect data at either 60-second epochs (in 2010) or 15-second epochs (in 2011). The GPS receivers were set to record the spatial coordinates of their location every five seconds. Participants were asked to wear these on an elastic waist belt during waking hours and to recharge them each night.

#### Travel diary

Participants completed a seven-day prospective travel diary (Appendix 1), in which they recorded the start and end time of each journey and all modes of travel used. The diary was closely based on that used in the UK National Travel Survey (Stratford et al., 2003).

### Sample

As in our previous method development paper (Panter et al., 2014), this analysis required objective locational data to be processed to identify commute journeys and times. We randomly selected a subsample of the 182 participants who had both objective and travel diary data on commuting in either 2010 or 2011, aiming to achieve a minimum of 50 journeys for the most commonly reported types of commuting journey in the sample. These were journeys made by car only; bus journeys, with or without walking or cycling to or from the bus stop; journeys made by car in combination with walking, or with cycling combined; and journeys made by walking only, or by cycling only. However, we were unable to obtain 50 journeys made only by walking because commuters living in the same immediate area of the city as their workplace had been excluded from recruitment to the cohort. We use the term ‘journey’ here to refer to the entire trip between home and work regardless of the number of modes of transport used. We use the term ‘mode of transport’ to refer to car, bus, cycling or walking, and ‘combination of modes’ to refer to the use of multiple modes within a journey, for example when a commuter drives from home to a park-and-ride facility and uses public transport, walks or cycles the remainder of the journey (Panter et al., 2013). To be included in analysis, participants had to provide (i) valid and complete GPS data reflecting usual journeys between home and work (see *Data processing*); (ii) synchronous GPS and AccHR data and complete travel diary information on at least three days; and (iii) plausible heart rate and acceleration values.

### Data processing

GPS data are increasingly used to study physical activity behaviour worldwide (Taylor et al., 1978) and as a result there are calls for standardised systems to process these data. A web-based application, known as PALMS, has been developed to process GPS data in a more standardised way and shown to be valid (Carlson et al., 2015). However, it currently requires data to be uploaded to a server held in the US. This may not be compatible with the Data Protection Act – the legislation that governs the use of personal data in the UK – which limits the export of identifiable data outside the European Economic Area. We therefore developed our own procedures which are described in detail here.

#### (i) Defining commute times on each journey from GPS data

GPS data were visually inspected in ArcGIS (version 10.0) to identify the start and end times for each journey to or from work. The start time was defined as the first five-second epoch after which participants left either their home or the outline of their workplace building, and the end time as the last five-second epoch before they reached the corresponding destination. Because journeys did not always begin at the start of a ‘clock minute’ (e.g. precisely at 10:00:00), the first and last clock minute of each journey were excluded to avoid misclassifying non-commuting activity as part of the journey. This ensured that metabolic cost data included in our calculations were drawn from the journey itself. We identified whether participants had travelled to or from work via an intermediate destination such as a school or shop (either visible on background mapping in ArcGIS, or reported in the travel diary) and remained there for more than five minutes without a reported change in mode. By examining GPS data, we were able to exclude the time spent at such intermediate destinations to more accurately reflect the metabolic cost of the journey itself. Both direct and indirect (‘via’) commuting journeys were included in analysis, to reflect the varied habitual commuting patterns of the study population. However, ‘via’ journeys were excluded if they included an intermediate destination more than 100 km from work or home. Further technical details of the processing and cleaning of GPS data are published elsewhere (Panter et al., 2014).

#### (ii) Extracting and processing energy expenditure data

Heart rate data were pre-processed (Stegle et al., 2008) using a simple individual calibration of heart rate based on sleeping heart rate, age and gender (Brage et al., 2007). Marginal metabolic cost was estimated using branched equation modelling (Brage et al., 2004) but translated to standard METs by adding 1 MET for each minute. We summarised AccHR data in one minute epochs and annotated each trace with the journey start and end times from GPS, as described above.

### Statistical analyses

Metabolic cost and distribution of time spent in sedentary behaviour (< 1.5 MET), light (1.5–3 MET), moderate (3–6 MET), and vigorous (> 6 MET) intensity physical activity, as well as moderate-to-vigorous physical activity (MVPA), were summarised for each mode or combination of modes of transport used on a journey using medians and interquartile ranges (IQR) because the data were not normally distributed within journeys. Ten bus commuters reported only the bus stages of their journeys and failed to report their access and egress modes, even though they may have walked or cycled to or from the bus stop. These bus journeys were grouped with those in which walking or cycling was reported as part of a bus journey.

Differences in metabolic cost between modes or combinations of modes of transport were assessed using mixed-effects linear regression, with metabolic cost as the continuous outcome variable, mode of transport as the categorical independent variable and adjustment for age and sex. This approach accounted for clustering of epochs (level 1) within journeys (level 2) and journeys within participants (level 3), and allowed the analysis to reflect the variation in metabolic cost within journeys on account of the stopping and starting (e.g. at traffic lights) and irregular speeds characteristic of everyday commutes. We checked the amount of variance in metabolic cost at the journey and participant levels with intraclass correlation coefficients by examining the random-effects parameters of an empty model, prior to running the final regression analysis. This showed that approximately 17% and 31% of the variance was represented at the journey and participant levels respectively, which further justified the use of a three-level mixed-effects linear regression model. Post-hoc Wald tests were conducted to assess differences between the beta coefficients for each transport mode. We chose to use linear models as the residuals were normally distributed.

### Sensitivity analysis

As processing GPS data is a time consuming technical process and collection of GPS data may raise some concerns amongst participants leading to drop-out we conducted a sensitivity analysis using journey start and end times reported by participants in their travel diaries to delineate journeys in AccHR traces. Our analyses assessed whether the resulting metabolic costs were comparable to those obtained from GPS-derived journey times using an adapted paired-sample signed-rank test, which accounted for clustering of journeys within participants (Newson, 2006).

## Results

### Participant and journey characteristics

Of 182 participants, 62 were randomly selected aiming to achieve a minimum of at least 50 journeys for the most commonly reported types of commuting journey in the sample. After excluding participants without valid GPS and synchronous AccHR data and complete travel diary information, this left 41 participants who made 182 journeys. These participants (56% women) were aged between 24 and 62 years (mean 46.2 years, sd 11.3). The majority reported having sedentary occupations (75%), had at least a bachelor's degree (85%) and had access to a car (93%). The median body mass index (BMI) was 24.5 kg/m^2^, 61% of participants were underweight or normal weight and 39% were overweight or obese (World Health Organisation, 2000). Participants lived in a mixture of urban areas (51%), towns and suburban areas (27%) and villages, hamlets or isolated dwellings (22%) (Bibby & Shepherd, 2004) and this was reflected in a range of commute distances (from GPS data: median 19 km, IQR 8 to 29 km).

**Fig. 1:**
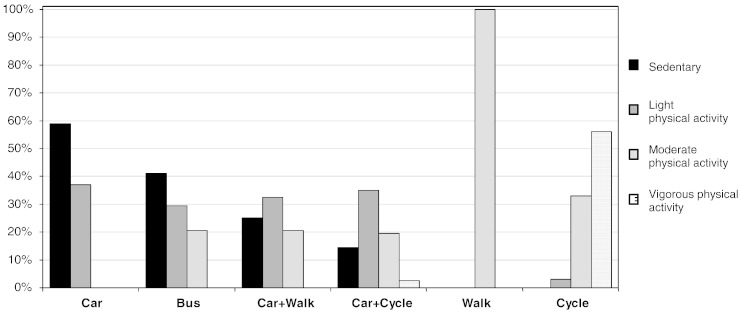
Median percentage of journey duration spent sedentary and in light, moderate, and vigorous physical activity. Note: This figure presents the median percentage of journey duration spent sedentary and in different intensities of physical activity because the data were highly skewed. As a result, the values of the columns within each journey type may not sum to 100%. Time spent in activity was classified as sedentary behaviour (< 1.5 MET), light (1.5–3 MET), moderate (3–6 MET), and vigorous (> 6 MET) intensity physical activity. Data were collected in 2010 and 2011 in Cambridge.Compared to those who drove all the way to or from work, participants using any other mode or combination of modes of transport recorded journeys of higher metabolic cost (Table 2; all *p* ≤ 0.04). For example, those using the bus expended an additional 0.7 MET on average, and those who walked or cycled all the way expended nearly an additional 2.5 and 3.9 MET respectively, than those who drove all the way.

Estimated metabolic costs for each mode or combination of modes of transport are shown in Table 1. For journeys in which walking and cycling were reported in combination with other modes, the median proportion of the journey duration spent in MVPA was 20% and the median proportion spent sedentary ranged from 15% to 41%. For car-only journeys, in contrast, the median time spent in MVPA was zero and the median proportion of the journey duration spent sedentary was 59% (Fig. 1).

#### Sensitivity analysis

Median metabolic cost estimated from GPS-derived journey times was on average 0.23 MET higher than those estimated from start and end times reported in travel diaries (*Z* > 6.17; *p* < 0.001).

## Discussion

### Principal findings

In this study, we have shown that GPS and AccHR data from free-living participants can be used simultaneously to provide objective estimates of the metabolic cost associated with a range of modes of transport for commuting. Whilst walking or cycling all the way to or from work involved higher metabolic cost as expected, their incorporation into longer motor vehicle journeys also made an important contribution. On average 20% of the duration of these multimodal journeys – equating to around 8 minutes for an average journey – was spent in physical activity of at least moderate intensity. Over the course of a working week, this form of active commuting could therefore amount to over half the recommended levels of physical activity for adults (Department of Health, 2011).

### Strengths and limitations

We assessed the metabolic cost associated with several modes and combinations of modes of transport in a sample of adult commuters living in both urban and rural settings. This represents a considerable improvement on existing laboratory studies using pre-set speeds (Ryschon and Stray-Gundersen, 1991, Shephard, 1982) or studies reliant on estimating speeds from self-reported time data (Ainsworth et al., 2011). Our use of GPS and combined acceleration and heart rate monitoring allowed us to collect continuous objective measurements over several days in the free-living environment, and ensured our results were robust to daily variations in speeds and distances. Whilst the modes of transport used for a given journey may have been susceptible to misreporting, the use of a day-by-day diary minimised this risk. With the exception of journeys made solely on foot, we were able to include journeys over a considerable range of both duration and distance. However, the setting of our study in Cambridge may limit the generalisablity of our findings to other settings, such as those with more varied topography where higher metabolic costs may be expected. Although a direct comparison with the local population is difficult because we recruited commuters from an area that was not coterminous with administrative boundaries, comparison with census data for working-age residents of Cambridge city and surrounding district council areas suggested that our sample contained a higher proportion of women, older adults and those with a degree, and a smaller proportion of those who rented their home and those aged 16–30 (Office for National Statistics, 2011). Concerns about data protection prevented us from using the web-based PALMS application for processing GPS data in this study, although it may be possible to overcome this limitation in future. We were also unable to assess individual fitness or take it into consideration in this study; individual calibration may provide more accurate estimates of metabolic rate at the individual level.

### Implications for research and practice

In our sample, incorporating walking or cycling into the commute always resulted in some journey time being spent in MVPA. When journeys were made by walking or cycling alone, a median of 89% or 100% of journey time respectively was spent in MVPA. When walking or cycling were combined with the use of motor vehicles, a median of 21–23% of the journey time was spent in MVPA. This equated to approximately 8 minutes per journey on average, which is around the advised minimum bout duration (Department of Health, 2011). Over the course of a working week, these walking or cycling stages of longer journeys would therefore contribute substantially to achieving the 150 minutes of MVPA recommended in current physical activity guidelines for adults (Department of Health, 2011). For example for journeys made by bus, taking the median duration of 45.5 minutes, with 20% of time spent in MVPA and assuming two journeys made on five working days, an individual would accumulate 91 minutes of MVPA, which equates to 60% of the adult physical activity guidelines. Higher intensity activity across the journey could also aid in the prevention or management of weight gain. For example, by substituting the last kilometre of a journey usually made by car (30 min at 1.28 MET/min) for a 10 min walk (thereby making a total journey of 40 min at 1.78 MET/min), this would result in an additional metabolic cost of 26 kcal per journey (or 52 kcal per day) for a 67 kg woman. Changes of this magnitude could be sufficient to offset weight gain in a proportion of the population (Hill et al., 2003). Strategies to promote this form of active commuting (e.g. through the use of park-and-ride facilities) may provide a feasible and affordable way of shifting population activity patterns, and would also be expected to have important health co-benefits by reducing air pollution and carbon emissions (Rabl & de Nazelle, 2012).

Our estimate of the metabolic cost of walking for commuting (4.6 MET) was slightly higher than that provided for walking to work in the physical activity compendium (Code 17270, 4 MET) (Ainsworth et al., 2011). However, our estimate for cycling (6.4 MET) was in line with that previously provided for bicycling at a self-selected pace (Code 01011, 6.8 MET), but higher than that for bicycling to work at < 10 mph (Code 01010, 4 METs). In our sample, the average speed for cycling on the commute was 10 mph and the distances travelled by cyclists were similar to those reported elsewhere (Hendriksen et al., 2000, Oja et al., 1991). Even in Cambridge, where cycling is relatively prevalent and normalised, we have shown that cycling at a self-selected speed is of sufficient intensity to meet physical activity recommendations. We found that estimates of the metabolic cost of journeys were slightly higher when using GPS-derived journey times than when using self-reported journey times. On the one hand, given that self-reported journey times are consistently over-reported (Kelly et al., 2013), this may be explained by participants having included travel-related activities such as waiting times, which may have lower energy expenditure than the travel itself, in their ‘travel’ times. On the other hand, it may reflect our decision to discard the first and last clock minute of each GPS-defined journey. This truncation was necessary to match all data to a common temporal unit for analysis and because not all journeys started at exactly the same time within an epoch. This decision will have modest consequences for the estimation of cumulative energy expenditure over the course of a week. Further research should aim to develop methods to automatically process and extract speed and elevation data from GPS devices which are essential for quantifying intensity of physical activity on the commute (Carlson et al., 2015, Ellis et al., 2014).

This study has provided more realistic estimates of the metabolic cost of commuting journeys under free-living conditions than previous studies reflecting the real behaviour of commuters, (Shephard, 2008, World Health Organisation, 2000) particularly for journeys in which walking or cycling are combined with the use of motor vehicles. These estimates show that incorporating active travel into the commute, however that is achieved, can contribute to meeting physical activity guidelines. The findings will help more accurate modelling of the health impacts of interventions to promote walking and cycling on the commute (Rabl & de Nazelle, 2012). Future longitudinal research should aim to confirm emerging evidence that switching to active transport is associated with an increase in overall physical activity without a commensurate decrease in recreational activity (Sahlqvist et al., 2013), and to assess the impact of incorporation of walking and cycling into the commute on obesity and other health outcomes.

## Conflict of interest statement

There are no conflicts of interest.

## Figures and Tables

**Table 1:** Metabolic cost of different modes or combinations of modes of transport on the commute.

Journey type	Sample size (journeys/individuals)	Median (IQR) journey duration (min)	Median (IQR) journey distance (km)	Median (IQR) intensity (MET)
Car	35/11	31 (22–38)	25.88 (20.50–29.63)	1.28 (1.16–1.78)
Bus	38/10	45.5 (38–65)	12.41 (8.21–22.85)	1.67 (1.16–2.02)
Car and walking combined	34/12	47 (33–65)	35.87 (28.67–40.26)	1.78 (1.56–2.57)
Car and cycling combined	28/15	35 (33–41)	20.71 (19.01–23.55)	2.21 (1.67–3.03)
Walking	15/5	14 (2–22)	1.41 (0.30–2.16)	4.61 (4.29–4.95)
Cycling	32/8	27 (14–42)	7.24 (3.71–12.13)	6.44 (4.40–7.00)

IQR — interquartile range; MET — standard metabolic equivalents. Journey duration and distance were derived from GPS data. Data were collected in 2010 and 2011 in Cambridge.

**Table 2:** Mixed-effects linear regression coefficients for metabolic cost (MET) of journey types.

Journey type	β	Standard error	95% CI	p	
Fixed effects					
*Reference: car-only*					
Bus	0.68*	0.30	0.10 to 1.26	0.024	
Car and walking combined	0.57*	0.23	0.12 to 1.03	0.013	
Car and cycling combined	1.58	0.34	0.91 to 2.25	< 0.001	
Walking	2.49	0.41	1.68 to 3.28	< 0.001	
Cycling	3.90	0.26	3.40 to 4.40	< 0.001	
Constant	1.65	0.57	0.54 to 2.78	< 0.001	

Random effects	Estimate	Standard error	95% CI		
sd(Constant) — journey level	0.51	0.04	0.44 to 0.59		
sd(Constant) — individual level	0.73	0.10	0.55 to 0.96		
sd(Residual)	1.36	0.01	1.33 to 1.38		

β — beta coefficient; CI — confidence interval; sd — standard deviation. *Wald tests *p* < 0.05 for all differences between coefficients, except between those for ‘Bus’ and ‘Car and walking combined’. Adjusted for age and sex. Data were collected in 2010 and 2011 in Cambridge.
