# Supplementary material for: Quantifying the physical activity energy expenditure of commuters using a combination of global positioning system and combined heart rate and movement sensors
Source: Prev Med. 2015 Dec;81:339–44. doi: 10.1016/j.ypmed.2015.09.022 (PMC4678256; doi:10.1016/j.ypmed.2015.09.022)
Supplement: Supplementary file 1 — Appendix 1 [file mmc1.docx]

**Appendix 1: Example travel diary for one day**

**1**

**DAY:**

**Household Member: A** *Please circle the day of the week****:* Mon Tue Wed Thu Fri Sat Sun Date _____________**

| **JOURNEYS: Please record each journey using a separate row and remember to tell us about return journeys** | | | | | | **STAGES: These columns are for entering details of each stage of your journey Only fill in the last two columns if you used a CAR or other MOTOR VEHICLE for any part of your journey** | | | | | | |
| --- | --- | --- | --- | --- | --- | --- | --- | --- | --- | --- | --- | --- |
|  | What was the purpose of your journey? *See Note A* | What time did you leave? *See Note B* | What time did you arrive? *See Note C* | Where did you start your journey? Tick ‘Home’ or give the name of the village, town or area. *See Note D* | Where did you go to? Tick ‘Home’ or give the name of the village, town or area. *See Note E* |  | What mode of transport did you use for each stage of your journey? *See Note F* | How far did you travel in miles? *See Note G* | How long did you spend travelling in minutes? *See Note H* | How many people travelled including you? *See Note I* | Which car or other motor vehicle did you use? *See Note J* | Were you the driver (D) or a passenger (P)? *See Note K* |
| 1 | *Go to work* | Time:  *8.00*  [✓] am  [ ] pm | Time:  *8.23*  [✓] am  [ ] pm | [✓] Home | [ ] Home  *Cambridge City Centre* | 1 | *Car* | *3.5* | *16* | *2* | *1* | [✓] D [ ] P |
|  |  |  |  |  |  | 2 | *Park & Ride Bus* | *3* | *15* | *2* |  | [ ] D [ ] P |
|  |  |  |  |  |  | 3 | *Walk* | *1* | *17* | *1* |  | [ ] D [ ] P |
| 2 | *Go home* | Time:  *4.30*  [ ] am  [✓] pm | Time:  *4.55*  [ ] am  [✓] pm | [ ] Home  *Cambridge City Centre* | [✓] Home | 1 | *Walk* | *1* | *16* | *1* |  | [ ] D [ ] P |
|  |  |  |  |  |  | 2 | *Park & Ride Bus* | *3* | *15* | *1* |  | [ ] D [ ] P |
|  |  |  |  |  |  | 3 | *Car* | *3.5* | *16* | *1* | *1* | [✓] D [ ] P |
| 3 | *Food shopping* | Time:  *7.30*  [ ] am  [✓] pm | Time:  *7.42*  [ ] am  [✓] pm | [✓] Home | [ ] Home  *Tesco, Bar Hill* | 1 | *Car* | *5* | *12* | *1* | *1* | [✓] D [ ] P |
|  |  |  |  |  |  | 2 |  |  |  |  |  | [ ] D [ ] P |
|  |  |  |  |  |  | 3 |  |  |  |  |  | [ ] D [ ] P |
| 4 | *Go home* | Time:  *8.45*  [ ] am  [✓] pm | Time:  *8.58*  [ ] am  [✓] pm | [ ] Home  *Tesco, Bar Hill* | [✓] Home | 1 | *Car* | *5* | *13* | *1* | *1* | [✓] D [ ] P |
|  |  |  |  |  |  | 2 |  |  |  |  |  | [ ] D [ ] P |
|  |  |  |  |  |  | 3 |  |  |  |  |  | [ ] D [ ] P |
| 5 |  | Time:  [ ] am  [ ] pm | Time:  [ ] am  [ ] pm | [ ] Home | [ ] Home | 1 |  |  |  |  |  | [ ] D [ ] P |
|  |  |  |  |  |  | 2 |  |  |  |  |  | [ ] D [ ] P |
|  |  |  |  |  |  | 3 |  |  |  |  |  | [ ] D [ ] P |

Notes

A. What was the purpose of your journey? Please give a simple description such as ‘go to work’, ‘take children to school’ or ‘go home’. If you went shopping please note whether it was ‘food shopping’ or ‘other shopping’.

B and C. What time did you leave (arrive)? Write in hours and minutes (e.g. 9:15). Please tick am or pm to show the time of day.

D and E. Where did you start your journey (go to)? Please write down the name of the place where your journey started and finished. If this was a large town or city give the name of the area. If you went to a shopping centre or visitor attraction please tell us its name. Please be as precise as possible. If your journey started or finished at home, you only need to tick ‘Home’.

F. What mode of transport did you use for each stage of your journey? Use a different line for the mode of transport you used for each stage of your journey (e.g. car, train, bus, bike). For example, if you drove to a Park & Ride site, caught a bus and then walked to your final destination, please tell us about the car, bus and walking stages.

G. How far did you travel in miles? Please give us the distance you travelled in miles, separately for each stage of your journey.

H. How long did you spend travelling? Please note the amount of time you spent travelling, separately for each stage of your journey. Do not include any time you spent waiting for public transport.

I. How many people travelled including you? Please write in the number of people, including yourself, who set out together. Only include people who were with you for at least half the distance of your journey.

J. Which car or other motor vehicle did you use? Please tell us the code number (e.g. ‘1’) of the vehicle if it is listed in the table at the front of this booklet. If you travelled in a vehicle not listed in the table, please tell us that (e.g. ‘Friend’s car’).

K. Were you the driver (D) or a passenger (P)? Please tick ‘D’ if you were the driver or ‘P’ if you were a passenger in the vehicle.

If you made more than six journeys on this day please use the extra space towards the back of the booklet.
